# T Cell Recognition of Tumor Neoantigens and Insights Into T Cell Immunotherapy

**DOI:** 10.3389/fimmu.2022.833017

**Published:** 2022-02-10

**Authors:** Malcolm J. W. Sim, Peter D. Sun

**Affiliations:** Laboratory of Immunogenetics, National Institute of Allergy and Infectious Diseases, National Institutes of Health (NIH), Rockville, MD, United States

**Keywords:** T cell receptor, tumor neoantigen, binding affinity, TCR and neoantigen bound HLA complex, group I and II neoantigens, adopt T-cell transfer immunotherapy, tumor infiltrating lymphocytes

## Abstract

In cancer, non-synonymous DNA base changes alter protein sequence and produce neoantigens that are detected by the immune system. For immune detection, neoantigens must first be presented on class I or II human leukocyte antigens (HLA) followed by recognition by peptide-specific receptors, exemplified by the T-cell receptor (TCR). Detection of neoantigens represents a unique challenge to the immune system due to their high similarity with endogenous ‘self’ proteins. Here, we review insights into how TCRs detect neoantigens from structural studies and delineate two broad mechanistic categories: 1) recognition of mutated ‘self’ peptides and 2) recognition of novel ‘non-self’ peptides generated through anchor residue modifications. While mutated ‘self’ peptides differ only by a single amino acid from an existing ‘self’ epitope, mutations that form anchor residues generate an entirely new epitope, hitherto unknown to the immune system. We review recent structural studies that highlight these structurally distinct mechanisms and discuss how they may lead to differential anti-tumor immune responses. We discuss how T cells specific for neoantigens derived from anchor mutations can be of high affinity and provide insights to their use in adoptive T cell transfer-based immunotherapy.

## Introduction

Immunotherapy is revolutionizing the treatment of cancer and understanding how the immune system detects tumors will lead to improved and novel therapies (1, 2). Tumor transformation is associated with a multitude of cellular and genetic changes including somatic mutations that alter protein sequence (3–5). Unleashing T cells specific for these mutations is thought to be one mechanism for the therapeutic effect of checkpoint blockade immunotherapy (CBI) (6, 7), and maybe the ‘common pathway’ for many effective immunotherapies (8). Extensive tumor genome and exome sequencing studies have revealed the landscape of tumor mutations to be broad, however only a fraction of these appear immunogenic (9, 10). Generating tools that can identify immunogenic neoantigens from sequence will greatly facilitate the deployment of neoantigen based vaccines and other immunotherapies (11). However, the features that distinguish immunogenic and non-immunogenic mutations are poorly defined. Structural biology has been invaluable to understanding the immune system (12–14). By studying TCRs with demonstrated clinical efficacy, structure-based approaches can provide insight into biochemical and structural features associated with therapeutic success (15). Here, we review recent structural studies of how TCRs detect immunogenic neoantigens and discuss how some biochemical properties, such as antigenic binding affinity, may influence clinical outcome of adoptive T cell therapy.

## Basics of T Cell Recognition and Antigen Presentation

### Antigen Presentation

**Figure 1:**
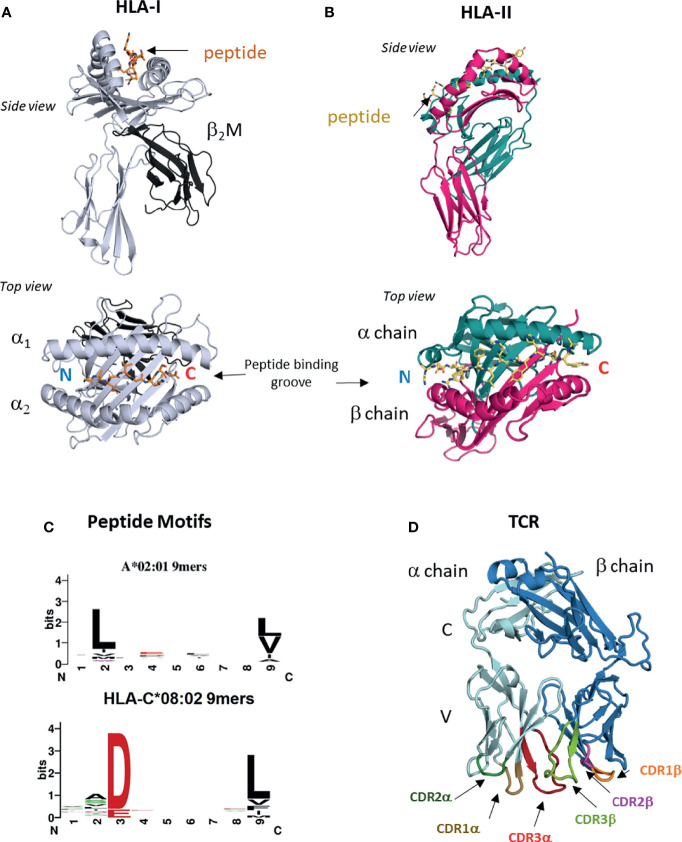
HLA-I, HLA-II and TCRs. **(A)** Structure of HLA I molecule. Contains HLA-I heavy chain, b_2_M and bound peptide. Peptide binding groove consists of a_1_ and a_2_ domains of HLA-I heavy chain (PDB entry 6ULI). **(B)** Structure of HLA-II molecule. Contains HLA-II alpha and beta chains and bound peptide. Peptide binding groove is shared by alpha and beta domains (PDB entry 1AQD). **(C)** Examples of peptide motifs for 9mer peptides eluted from two different HLA-I molecules, HLA-A*02:01 and HLA-C*08:02. Single letter amnio acid code is used. Size of letter indicates prominence of that residue. Anchor residues are defined by restricted amino acid usage, commonly at p2, p3 and p9. **(D)** Structure of ab TCR showing constant (C) and variable (V) regions and six CDR loops (PDB entry 6ULN).

T cell recognition is a multi-step process that includes two steps where structural biology can provide unrivaled insight. The first is antigen presentation, where peptide antigens are presented on the cell surface on class I or class II human leukocyte antigens (HLA) (16). HLA-I is expressed on all nucleated cells, including tumors and is the ligand for TCRs expressed on CD8^+^ cytotoxic T cells. Humans carry three classical HLA-I genes, encoded by *HLA-A*, *HLA-B* and *HLA-C* that encode the HLA-I heavy chain, which forms the HLA-I molecule in complex with bound peptide and the invariant chain beta-2-microglobulin (β_2_M) (**Figure 1A**). HLA-I bound peptides are typically 8-11 amino acids long, due to a closed peptide binding groove that prevents longer peptides from extending at either termini. At homeostasis, HLA-I binds ‘self’ peptides, which are derived from the proteasomal degradation of old proteins (retirees) or the products of stalled ribosomal translation known as defective ribosomal products (DRiPs) (17, 18). These peptides are then funneled into the ER by the transporter associated with antigen presentation (TAP) (16, 18), where they are loaded onto HLA-I molecules in a competitive manner facilitated by chaperone proteins such as TAPASIN (19). In infections or cancer, pathogen derived sequences or neoantigens enter the HLA-I presentation pathway the same way as ‘self’ peptides in the form of ‘retirees’ and DRiPs (17, 18, 20).

The ligand for TCRs expressed by CD4^+^ helper T cells is HLA-II, which is expressed on professional antigen presenting cells (APC) such as dendritic cells, monocyte/macrophages and B cells. HLA-II consists of bound peptide and two chains alpha and beta encoded by different polymorphic genes (**Figure 1B**). HLA-II bound peptides are typically 15 amino acids in length, longer than HLA-I due to its open-ended peptide binding groove (PBG). There is some evidence that tumors can directly express HLA-II (21) but generally, recognition of HLA-II restricted neoantigens is thought to be through interactions with APCs (22). HLA-II binds peptides in a late endosomal compartment where it intercepts endocytosed proteins, which are degraded by endosomal proteases (16). Chaperones such as HLA-DM facilitate exchange for high affinity peptides (23). Neoantigens can enter this pathway *via* endocytosis of apoptotic or necrotic tumor cells bearing specific mutations.

The genes encoding classical HLA molecules are the most polymorphic across human populations, with over 10,000 HLA-A, -B, -C and 5,000 HLA-II protein variants (24, 25). The majority of this polymorphism is located within the PBG, a specialist structural fold that allows HLA proteins to bind peptides of correct sequence *via* non-covalent interactions. As peptide binding to HLA is highly competitive, only peptides that best satisfy the biochemical requirements of the particular PBG will escape quality control and be presented on the cell surface (19). The PBG is made of pockets (A-F) that exhibit localized preferences for specific biochemical characteristics, such as charge, size, hydrophobicity, polarity and combinations of all (26). The A-F pockets run from the peptide N to C termini, with the A and F pockets coordinating the conserved amide and carboxylic acid groups. For HLA-I binding, there are critical residues at p2 or p3 and the C-terminus (pΩ) positions, known as anchor residues. Amino acid substitutions at anchor residues substantially alter peptide binding and HLA stability. The fraction of the proteome bound by HLA molecules is known as the immunopeptidome (27), and these peptides can be eluted, and sequenced by mass spectrometry (28–31). Immunopeptidomes are highly diverse consisting of hundreds to thousands of different ‘self’ peptides for each HLA allotype. Allotype-specific peptide motifs are derived from immunopeptidomes of different HLA-I and HLA-II molecules and demonstrate peptide sequence restriction at anchor residues and variation at non-anchor residues (**Figure 1C**). Crystal structures of HLA-I and HLA-II molecules with specific peptides confirm these motifs by revealing the number of interactions between the PBG and peptide anchor side chains. It is possible to classify tumor mutations into three categories; (1) mutation occurs at a non-anchor residue of an existing ‘self’ peptide, (2) mutation occurs at anchor residue impacting antigen presentation, (3) mutation falls in a peptide sequence not presented by host HLA allotype. Herein we use nomenclature defined by Fritsch et al. (32); group 1 neoantigens exhibit similar HLA binding affinities between mutant and ‘wild type’ (WT) peptides and the mutation lies in a non-anchor residue in an existing ‘self’ peptide. Group 2 neoantigens exhibit significantly increased HLA binding affinity compared to WT peptides due to mutations that form novel anchor residues.

### T Cell Recognition

Once presented by HLA-I and HLA-II molecules, neoantigen peptides can be detected by T cells *via* the alpha-beta T cell receptor (αβTCR). TCR α and β chains consist of constant (C) and variable (V) regions, where the membrane distal V regions engage peptide-bound HLA complexes (**Figure 1D**). From structural studies of TCR : HLA complexes, general rules have emerged (33, 34). The V regions contact peptide and HLA *via* three complementarity determining regions (CDR1-3) that form six flexible loops, generated through VDJ recombination (33, 34). Germline encoded CDR1 and CDR2 engage the HLA protein, while CDR3s interact with bound peptide. The TCR Vα is centered over the α2 helix of HLA-I (the β chain of HLA-II) while the TCR Vβ chain is centered over the α1 helix of HLA-I (the α chain of HLA-II). The TCR docks diagonally, with the Vα angled towards the peptide N-terminus and the Vβ angled towards the peptide C-terminus. Substitutions that disrupt key CDR interactions with peptide or HLA are sufficient to reduce or eliminate binding and prevent T cell activation (33–35). During T cell development in the thymus, Vα and Vβ chains are generated by recombining single V(D)J gene segments from a large repertoire of V, D and J segments (only V and J for α chain) (36, 37). Single gene segments recombine to form each chain allowing for considerable Vα and Vβ diversity, which when combined to form αβ pairs have a theoretical upper limit of over 10^15^ unique TCRs. V gene segments encode CDR1 and CDR2 sequences, while CDR3 is located at the V(D)J boundary and due to base editing is the most variable region. During T cell development the pre-selection TCR repertoire is pruned to eliminate TCRs with the potential for autoreactivity, while also selecting for useful TCRs with the ability to detect ‘self’ HLA. Before leaving the thymus, each TCR is selected for moderate affinity for ‘self’ peptide-bound HLA complexes (positive selection), while TCRs with too high affinity for ‘self’ peptide-bound HLA are deleted (negative selection) (37, 38). Consequently, many TCRs that recognize group 1 neoantigens may be eliminated due to their high similarity with WT ‘self’ peptides.

## Two Ways to Detect Neoantigens

**Figure 2:**
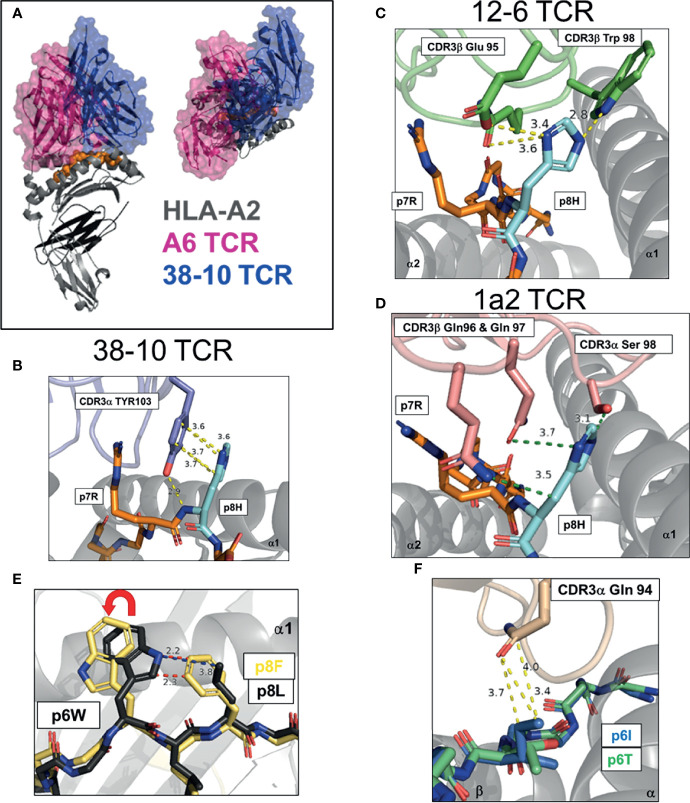
Multiple strategies to detect mutated ‘self’ epitopes, group 1 neoantigens. **(A)** Docking of A6 TCR and 38-10 TCR on HLA-A2 displaying C-terminal shift of 38-10 TCR specific for p53 R175H (PDB entry 1AO7, 6VRN). **(B–D)** Dominant TCR contacts with p8 of HMTEVVR**H**C, p53 R175H neoantigen presented by HLA-A2 by 38-10 TCR (6VRN) **(B)**, 12-6 TCR (6VRM) **(C)** and 1a2 TCR (6QVO) **(D)**. **(E)** Peptide pre-organization confers structural dissimilarity. Peptide p8 in HHAT L75F neoantigen KQWLVWL**F**L pre-organizes p6W into optimal TCR binding confirmation to allow effective tumor detection (6UK2,6UK4). **(F)** Direct recognition of exposed mutation. Mutant p6I in GELIG**I**LNAAKVPAD TPI neoantigen confers more TCR-E8 contacts that WT p6T (2IAM, 2IAN).

**Figure 3:**
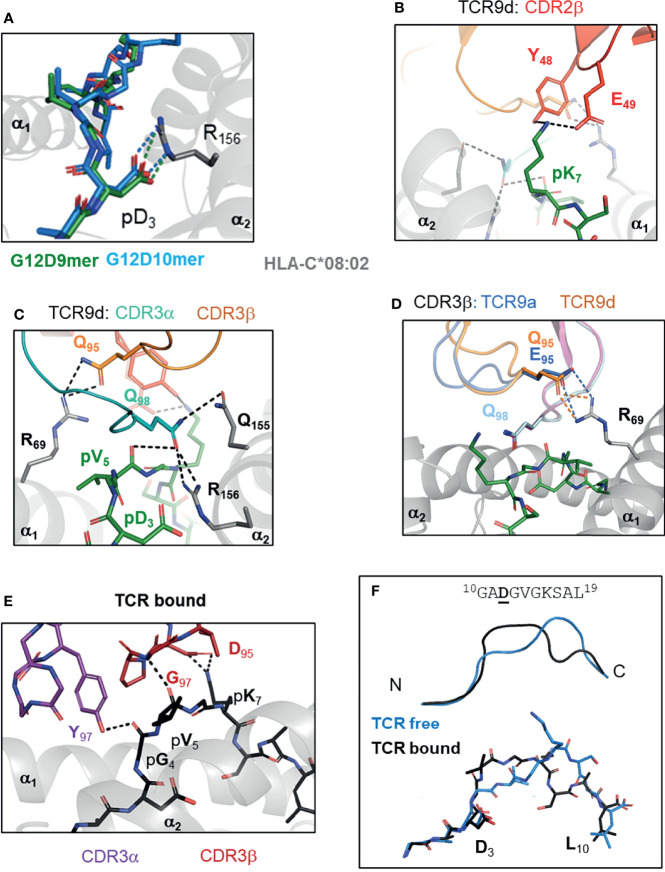
TCR recognition of group 2 neoantigens, novel ‘non-self’ epitopes generated by the KRAS-G12D mutation. **(A)** KRAS-G12D neoantigens G12D-9mer (GA**D**GVGKSA) and G12D-10mer (GA**D**GVGKSAL) form a salt bridge mutant p3 Asp and HLA-C Arg 156 on α2 helix of HLA-C*08:02 (PDB entry 6ULI, 6ULK). **(B-D)** TCR recognition of G12D-9mer by TCR9a and TCR9d. **(B)** Shared CDR2β contacts with p7 Lys (6ULN). **(C)** Shared CDR3α contacts with p5 Val and Gln 155, Arg 156 of HLA-C*08:02 (6ULN). **(D)** Position 95 CDR3b contact with Arg 69 of HLA-C*08:02 modifies TCR9 binding strength (6ULN,6ULR). **(E, F)** TCR10 recognition of G12D-10mer. **(E)** TCR10 CDR3α and CDR3β contacts with G12D-10mer (6UON). **(F)** G12D-10mer conformation in TCR free and TCR10 bound forms (6ULK,6UON).

**Figure 4:**
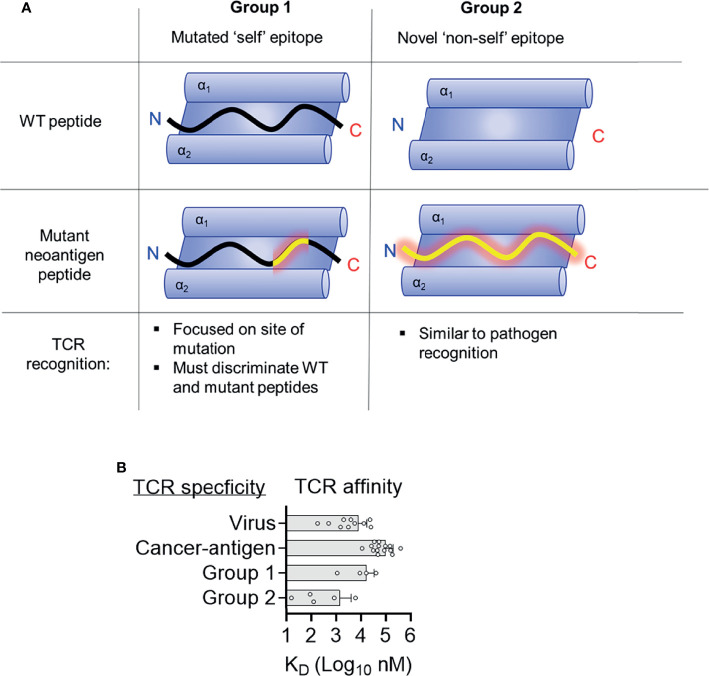
Two distinct structural mechanisms for detection of neoantigens. **(A)** Distinct features of antigen presentation and TCR recognition of group 1 and group 2 neoantigens **(B)** Affinities of TCRs specific for tumor antigens, viruses, and group 1 and group 2 neoantigens. Data for viruses and tumor antigens are from Aleksic et al. (44).

Insights from structural and functional studies have revealed that neoantigens can be recognized in two fundamentally different ways. Group 1 neoantigens are those where the mutation occurs in a non-anchor residue of an existing ‘self’ peptide (**Figure 2**). Group 2 neoantigens are where the mutation creates an anchor residue converting a previously non-HLA binding sequence into a novel non-self epitope (**Figure 3**). From the examples below, we review how there are multiple ways for TCRs to solve the problem of identifying single amino acid substitutions in pre-existing ‘self’ peptides (group 1) (39–41). Next we review our own work on the presentation and T cell recognition of two group 2 neoantigens derived from the same G12D mutation in the oncogene KRAS (15). To define neoantigens as group 1 or group 2, researchers often utilize prediction algorithms such as NetMHCPan to predict the HLA binding affinities of WT and mutant peptides (42). For group 1 neoantigens the predicted binding affinities will be similar, while for group 2 neoantigens the mutant peptide has considerably higher binding affinity than the WT sequence. For detailed studies of specific neoantigens, it is important to validate whether neoantigens fall into group 1 or 2 using *in vitro* assays such as peptide loading on TAP-deficient cells or *in vitro* refolding assays with recombinant proteins (15, 43). **Table 1** lists the HLA restrictions, neoantigen sequences and TCR affinities for the TCRs we reviewed. A summary of differences between TCR recognition of group 1 and group 2 neoantigens is shown in **Figure 4**.

**Table 1:** Neoantigen specific TCRs with crystal structures.

TCR	HLA	Peptide sequence		Protein	Mutation position	TCR affinity (KD)	
		WT	Mut			WT	Mut
**12-6**	A*02:01	HMTEVVRRC	HMTEVVR **H** C	p53	175	UD	1.1 μM
**38-10**	A*02:01	HMTEVVRRC	HMTEVVR **H** C	p53	175	UD	39.9 μM
**1A2**	A*02:01	HMTEVVRRC	HMTEVVR **H** C	p53	175	UD	16.2 μM
**302TIL**	A*02:06	KQWLVWLLL	KQWLVWL **F** L	Hedgehog acyltransferase	75	200 μM	9 μM
**E8**	DR1	GELIGTLNAAKVPAD	GELIG **I** LNAAKVPAD	Triosephosphate isomerase	28	ND	ND
**G4**	DR1	GELIGTLNAAKVPAD	GELIG **I** LNAAKVPAD	Triosephosphate isomerase	28	ND	ND
**TCR9a**	C*08:02	GAGGVGKSA	GA **D** GVGKSA	KRAS	12	NA	16 nM
**TCR9b**	C*08:02	GAGGVGKSA	GA **D** GVGKSA	KRAS	12	NA	835 nM
**TCR9c**	C*08:02	GAGGVGKSA	GA **D** GVGKSA	KRAS	12	NA	90 nM
**TCR9d**	C*08:02	GAGGVGKSA	GA **D** GVGKSA	KRAS	12	NA	125 nM
**TCR10**	C*08:02	GAGGVGKSAL	GA **D** GVGKSAL	KRAS	12	NA	6 μM

WT, wild type; MUT, mutant; UD, Undetectable; ND, Not determined; NA, Not applicable. ‘*’ is part of nomenclature for HLA alleles. Bold & underline indicate site of mutation.

## Group 1 Neoantigens: Mutated ‘Self’ Epitopes

### Recognition of a Shared Mutated p53-R175H Neoantigen by C-Terminal Shift

*TP53* is the most mutated gene across all cancers, highlighting its critical role as a tumor suppressor (45). A significant number of *TP53* mutations occur in the same ‘hotspot’ locations. Studies by the Rosenberg group at the National Cancer Institute (NCI) and others, have identified TCRs specific for the p53-R175H mutation, restricted by the common HLA-I allotype, HLA-A*02:01 (HLA-A2) (46–49). Approximately 5% of p53 mutations are R175H, and the high frequency of HLA-A2 across populations makes this ‘shared’ neoantigen an attractive therapeutic target (50). In a recent study by Wu et al, three p53-R175H specific TCRs were studied, 12-6, 38-10 and 1a2 (39). These TCRs displayed no binding to WT p53 peptide but a range of affinities (K_D,_ 1 - 40 μM) for the p53-R175H peptide (HMTEVVR **H** C). As this mutation occurred at a non-HLA-A2 anchor position, the authors were able to solve structures of HLA-A2 with both WT and R175H p53 peptides. The peptide conformations in these HLA-A2 alone structures were identical, apart from the peptide position 8 (p8) side chain. The TCRs shared no Vα or Vβ genes and their CDR3 sequences had no obvious sequence homology, suggesting each TCR recognized the p53-R175H mutation in a different way. By solving x-ray crystal structures of all three TCR-A2-p53-175H complexes, Wu et al. were able to answer this question directly. These TCRs displayed a canonical diagonal docking orientation but were unusually shifted to peptide C-terminus, proximal to the mutation site (**Figure 2A**). Indeed, TCR 38-10 was the most C-terminal shifted TCR in the PDB database with a canonical orientation, with TCR 12-6 and 1a2 also high on the list (39). By shifting towards the C-terminus, these TCRs were able discriminate between WT and mutant p53 peptides as most TCR-peptide contacts with p7-Arg and p8-His, while most TCRs target the central residues p4-p6 (33). Each TCR had different footprints, but 12-6 and 1a2 were more similar and utilize their CDR3β to coordinate the R175H mutation, while 38-10 was dominated by its CDR3α. For TCR 38-10, CDR3α Tyr103 slides between p7R and p8H to contact the peptide backbone, while forming π−π stacking interactions with the imidazole ring of p8H and van-der Waals contacts with p7R (**Figure 2B**). For TCR 12-6, the p8H side chain is contacted by CDR3β Glu95 and Trp98, while CDR3β Gln99 contacts the peptide backbone (**Figure 2C**). For TCR 1a2, p7R is coordinated by CDR3β Asp100 and CDR1a Tyr32, while p8H is contacted by CDR3β Gln96 and Gln97 with further contacts from CDR3α Ser98 (**Figure 2D**). Further contacts with the HLA-A2 heavy chain and modest conformational changes were observed to fully accommodate TCR binding (39). This study highlights how three different TCRs utilize different contacts but the same broad strategy of C-terminal shift to coordinate the same mutation.

### Structural Dissimilarity *via* Peptide Pre-Organization

In another recent study, a completely different structural solution to neoantigen recognition was observed (40). This study focused on an HLA-A*02:06 restricted TCR (302-TIL) specific for a L75F mutation in hedgehog acyltransferase (HHAT) identified from a patient with ovarian cancer (40). Similar to p53-R175H, the mutation was located at p8 of a 9mer antigen with the sequence KQWLVWL **F** L. However, unlike recognition of R175H, TCR recognition was not dependent on a C-terminal shift. In fact, the relatively conservative pL8F mutation, forces p6W into an optimal TCR binding conformation due to proximity with the larger p8F (**Figure 2E**). Crystal structures of 302-TIL TCR in complex with both WT *and* mutant peptide, revealed that the p6W adopted the same conformation in both complexes. However, 302-TIL TCR had a much higher affinity for mutant peptide at 9 μM compared to 200 μM with WT peptide and had an especially slower off rate with mutant peptide. The best interpretation of these data are that adopting the optimal p6W conformation is slow in the context of WT peptide, but p8F ‘pre-organizes’ p6W into an optimal TCR binding state, allowing rapid T cell activation and discrimination of tumors from healthy cells.

### Direct Recognition of Exposed Mutation

To date, there is one structural study of an HLA-II restricted neoantigen specific TCR identified from a melanoma specific CD4+ TIL cell line TIL1558 (41). TCR-E8 was identified as HLA-DR1 restricted and specific for a Thr28Ile mutation in the enzyme triosephosphate isomerase (TPI), with the peptide GELIG **I** LNAAKVPAD. TCR-E8 tetramers displayed binding only to mutant TPI-T28I peptide but not WT peptide by surface plasmon response (SPR), but the binding was too weak to determine a K_D_ using TCR monomers. The pT6I substitution had no impact on peptide stability of HLA-DR1 and thus was likely recognized due to novel TCR contacts. Complex structures of TCR-E8 with HLA-DR1 bound to WT and mutant TPI peptides were solved. In the complex with WT peptide, p6Thr is buried and forms only one TCR contact. In contrast, the mutant p6Ile is exposed and protrudes from the HLA-DR1 surface forming three TCR contacts, providing a higher buried surface area and improving the shape complementarity between TCR and HLA-DR1 (**Figure 2F**).

## Group 2 Neoantigens: Novel ‘Non-Self’ Epitopes Generated by Anchor Residue Mutations

The examples above involve direct comparisons between WT and mutant peptides as the mutations did not occur in anchor residues and had minimal effects on HLA stability. Group 2 neoantigens are peptides for which mutations create an anchor residue required for HLA binding and thus these epitopes acquired HLA presentation through mutation. In these cases, the WT peptides are generally not presented by HLA for exactly the opposite reason that they lack the right anchor residues for HLA binding. In theory, these epitopes appear as entirely ‘non-self’ and completely novel to the immune system analogous to pathogen derived peptides. They form specific interactions with their cognate TCRs and generate robust T cell response (15, 51).

### KRAS-G12D Mutation Creates Two HLA-C*08:02 Restricted Neoantigens

Oncogenic mutations in the RAS family of small GTPases (K-, N-, H-RAS) are second only in frequency to those in *TP53* (20, 52, 53). These mutations occur in ‘hotspots’ at positions 12, 13 and 61 of RAS protein and lead to constitutive RAS activation promoting tumor transformation (20, 54). The high frequency of these mutations makes them attractive targets for immunotherapy. In 2016, a seminal study demonstrated that adoptive transfer of expanded TILs specific for Gly 12 to Asp mutation in KRAS (KRAS-G12D) lead to tumor regression in a patient with metastatic colorectal cancer (51). Adoptive transfer of expanded TILs specific for KRAS-G12D led to complete regression of all but one metastatic lesion. The remaining lesion lost HLA-C*08:02 from its genome demonstrating that clinical efficacy in this case was most likely through HLA-C*08:02 presentation of KRAS-G12D neoantigens (51). Four HLA-C*08:02 restricted KRAS-G12D specific TCRs were identified from this study in addition to one more identified in a 2015 study (55). Of these five TCRs, four were KRAS-G12D 9mer specific (G12D-9mer; GA**D**GVGKSA), while one was KRAS-G12D 10mer specific (G12D-10mer; GA**D**GVGKSAL). The G12D-9mer specific TCRs are TCR9a, 9b, 9c & 9d, while TCR10 is the G12D-10mer specific TCR. For both neoantigens, the mutation occurred at Gly 12 of KRAS resulting in Aspartate at peptide position three. The G12D-10mer differs from G12D-9mer by having one additional Leu at the C-terminus, the next residue in the KRAS sequence.

By solving crystal structures of HLA-C*08:02 with G12D-9mer and G12D-10mer alone and in complex with cognate TCRs, we were able to directly assess the impact of G12D mutation on HLA-C binding and T cell recognition (15). Both G12D-9mer and G12D-10mer bind HLA-C*08:02 *via* a salt-bridge formed between p3 Aps and Arg156 of the α_2_ helix of HLA-C*08:02 (**Figure 3A**). This salt-bridge is a critical anchor interaction required for all HLA-C*08:02 binding peptides as evidenced by the fact that 97.6% of peptides eluted from HLA-C*08:02 had either Asp or Glu at p3 (28, 29). In contrast, the WT peptide with p3 Gly cannot form a salt-bridge with the HLA-C*08:02 and consequently only G12D KRAS peptides stabilized HLA-C*08:02 on cells or as recombinant protein (15). Thus, the G12D mutation generates a novel anchor interaction endowing mutant but not WT KRAS peptides to bind HLA-C*08:02 and be presented for immunosurveillance.

Class I HLA binding peptides contain a C-terminal anchor, the side chain of which is orientated to toward the interior of HLA peptide binding grove (F pocket) and is buried from solvent (26, 56). The C-terminal residue is typically large, hydrophobic or charged and interacts with a complementary hydrophobic or charged F pocket (26, 28, 56). The G12D-9mer contains an unusual C-terminal anchor (Ala), which does not fully occupy the F-pocket (15). Despite this, G12D-9mer bound HLA-C*08:02 with canonical conformation with p9 Ala positioned into the class I hydrophobic pocket (15). The most common C-terminal residue for HLA-C*08:02 bound peptides is Leu, however Ala is present in approximately 1% of peptides (57). Consistent with Ala being sufficient but not an optimal C-terminal anchor, substitution of G12D-9mer p9 Ala to Leu improved binding to HLA-C*08:02 and T cell recognition by TCR9a (15, 57). In the G12D-10mer, its canonical C-terminal anchor and the salt-bridge with p3 Asp, forced the G12D-10mer to bulge peaking at p7 Lys, adopting an entirely different conformation than G12D-9mer (15). This resulted in two HLA-C*08:02 bound G12D neoantigens, with distinct peptide conformations even though their sequences differed by only one amino acid.

TCR recognition of KRAS-G12D peptides contained the general features observed in many of TCR : HLA complexes (33). However, the unique aspects of KRAS-G12D specific TCRs provided insights to how tumor infiltrating T cells recognize tumors and the potential beneficial traits for selecting therapeutically effective TCRs (15). Consistent with their distinct peptide conformations, the G12D-9mer and G12D-10mer specific TCRs used different Vα and Vβ genes and shared no CDR sequences (15, 51). The four G12D-9mer specific TCRs (TCR9a, 9b, 9c and 9d) used the same Vα and Vβ genes, with almost identical CDR3α sequences despite the fact that TCR9d was identified from a different individual to the other three TCRs, suggesting TCR9 is a public TCR (51, 55, 58). The four G12D-9mer specific TCRs displayed a range of high affinities, from 16 nM (TCR9a) to 835nM (TCR9b) (15). TCR9 docks onto peptide:HLA-C as a rigid body without significant changes in peptide nor HLA-C conformation and most peptide contacts are made through CDR3α and CDR2β residues, conserved across TCR9a-d (**Figures 3B, C**). CDR3β is the only segment with significant sequence variation among TCR9a-d. Structures of TCR9a and TCR9d with HLA-C*08:02-G12D-9mer revealed the same contact with CDR3β position 95 and HLA-C Arg 69 on the α_1_ helix (15). The biochemical strength of the CDR3β-HLA-C interaction correlated with TCR9 affinity, with TCR9a Glu95 forming a salt-bridge and conferring the strongest binding. TCR9c and 9d formed h-bonds with HLA-C Arg 69 *via* Gln 95 and had intermediate affinities, while TCR9d could not contact HLA-C Arg 69 *via* Arg 95 in its CDR3β and consistently had the lowest affinity (15) (**Table 1**). Recognition of the G12D-10mer by TCR10 was dependent on CDR3α and CDR3β interactions with the central core of peptide (**Figure 3D**). Interestingly, TCR10 binding induced a conformational change in G12D-10mer peptide that is different from the peptide conformation presented by HLA-C*08:02 in the absence of the receptor (15).

G12D-9mer and G12D-10mer share VGK (p5-p7) exposed to TCR, however their discrete conformations resulted in distinct TCR contacts without any detectable conservation between the G12D-9mer and -10mer specific TCRs (15). For example, both TCR9 and TCR10 formed charge interactions with the peptide Lys at position 7, TCR9 used a Glu residue from CDR2β and TCR10 used an Asp residue from its CDR3β, respectively, to facilitate the charge contacts (15). TCR9a-c and TCR10 were identified in the same individual and the lack of conservation in V-gene and CDR3 sequences supports the conformational uniqueness of these neoantigens. Indeed, there is limited cross reactivity between the two classes of TCRs (15, 51). TCR10 is solely G12D-10mer restricted, exemplifying the conformational dependency of its interaction with G12D-10mer. TCR9a displayed weak recognition of G12D-10mer and structural modeling suggests TCR9a could interact with G12D-10mer in its TCR unbound conformation (15). However, it is also possible that G12D-10mer degrades during *in vitro* assays to confer weak recognition by TCR9, as we did not observe any TCR9 binding to G12D-10mer using recombinant protein (15). Together, our study demonstrated that the G12D-9mer and G12D-10mer are structurally distinct, prototypical group 2 neoantigens, which are recognized by their cognate TCRs *via* discrete mechanisms.

## Do T Cell Responses Differ Between Mutated ‘Self’ Epitopes and Novel ‘Non-Self’ Epitopes?

Seminal studies indicated that clinical responses to check-point blockade were associated with mutational burden (6, 59). Further investigations have sought to identify classes of mutations that best associate with clinical success, with a focus on neoantigen quality not quantity (44, 60, 61). Multiple studies suggest that neoantigens derived from anchor mutations (group 2) are more likely to be immunogenic and higher loads of anchor mutation neoantigens are associated with better prognosis than merely high numbers of neoantigens (62–64). One explanation for this is that group 2 neoantigens engender better T cell immunity than group 1 neoantigens, however molecular mechanisms for this effect are unclear. Group 2 neoantigens are more dissimilar to ‘self’ peptides compared to group 1 and there is evidence that T cell responses to HIV are better to those peptides most dissimilar to ‘self’ peptides (65). Similarly, neoantigens generated from novel open reading frames derived from insertion-deletion mutations (indels) are highly immunogenic and clearly dissimilar for WT ‘self’ sequences (66). A potential mechanism is that group 2 neoantigen specific TCRs are of higher affinity, allowing stronger T cell responses. There are a limited number of biophysical studies describing the affinities of neoantigen specific TCRs making it difficult to draw broad conclusions. However, neoantigen specific TCRs have similar affinities to viral specific TCRs, all of which are much higher affinities than TCRs specific to those tumor antigens, which are ‘self’ peptides with dysregulated gene expressions in tumors (**Figure 4B**) (67). TCRs specific for group 2 neoantigens were higher affinity than those specific for group 1 (**Table 1** and **Figure 3B**). One explanation for this trend is that high-affinity TCRs for group 1 neoantigens are likely deleted in the thymus owing to cross reactivity to ‘self’ antigens. In contrast, WT peptides from group 2 neoantigens are likely not to be presented in the thymus and therefore high-affinity TCRs can survive negative selection, exemplified by TCR9a with an affinity of 16 nM (15). It is important to stress however that data on neoantigens specific TCRs are limited and that the only group 2 neoantigen specific TCRs studied to date are HLA-C restricted, while the group 1 neoantigen specific TCRs are HLA-A restricted, which may impact their intrinsic affinities. Further studies of other neoantigen specific TCRs are needed to determine if this trend towards higher affinity in group 2 specific TCRs is maintained. It appears that intermediate affinity TCRs with low micromolar to high nanomolar affinities such as TCR10, 9b and 9c, are ideal for effective TCR therapy (68–70).

## Insights Into Selecting TCRs for Immunotherapy

Adoptive T cell transfer-based immunotherapy is a promising new approach to eliminate metastatic cancers. It assumes that many tumor infiltrating T cells (TIL) recognize tumor antigens specifically, but their circulating numbers are low in patients, thus natural TILs are insufficient to eradicate tumor cells. These TILs, however, can be expanded *in vitro* to large numbers and reinfused into cancer patients. The challenge is to know the right type of T cells to choose to expand into therapeutic reagents. Currently, the choice of T cells for expansion remains largely a trial-and-error empirical approach. Similar unknowns apply to ‘off the shelf’ TCR based therapies that transduce T cells with tumor specific TCRs. In principle, there are three main criteria for the selection of anti-tumor T cells: affinity, antigenic breadth and persistence. In the case of treating a metastatic colorectal cancer with adoptive transfer of expanded TILs specific for KRAS (KRAS-G12D) neoantigen presented by HLA-C*08:02, the transferred CD8+ T cells consists of four clonotypes, bearing TCR9a, 9b, 9c and TCR10, at abundance of 49.5%, 6.9%, 0.04% and 19.1% of the total transferred T cells, respectively. The treatment resulted in regression of all metastases that retained HLA-C*08:02 expression (51). Intuitively, high affinity receptors are more desirable in effective tumor killing, however high affinity TCRs appear to have diminished antigen sensitivity *in vitro* and *in vivo* (68–72). Another major concern of high affinity engineered TCRs is the potential for cross-reactivity, which can be lethal (73). However, this is unlikely to be a problem for naturally occurring high affinity TCRs specific for group 2 neoantigens such as TCR9a. Indeed, our data suggest that TCR9a and TCR10 have similar antigen sensitives with TCR9a being slightly more sensitive than TCR10 (15, 51, 57). The problem with clinical use of TCR9a is that despite an abundant initial presence of the highest affinity receptor (50% of the infusion), TCR9a was undetectable in the periphery on day 40 of post-transfer (15, 51). In contrast, the other three TCRs engrafted, resulting in a near inverse correlation between TCR affinity and their *in vivo* persistence (15, 51). In particular, TCR10 had the lowest affinity (6 μM), made up 20% of the infusion and was maintained in the periphery at 10% of the repertoire 9 months post-transfer. The T cells with high affinity receptors disappeared faster in circulation during the adoptive transfer therapy (15), while lower affinity T cells persisted longer. Given the vast number of TCR9a^+^ cells transferred (≈7×10^10^) it seems highly unlikely that TCR9a^+^ cells disappeared by decay, while the three other TCRs engrafted (51). A more likely scenario is that higher affinity T cells engage more effectively to cognate antigen (G12D-9mer) on the tumor resulting in higher tendency of activation induced cell death (AICD) rather than proliferation (68). It is not clear if all four TCR clonotypes were necessary for effective therapy in this case. In particular, was tumor recognition by the high affinity TCR, necessary for tumor regression? It is possible that the infusion of multiple TCRs specific for different antigens with a blend of characteristics including high affinity and long-term persistence was essential for effective tumor clearance. In this model, due to a ‘cold’ immune environment pre-infusion, a high affinity receptor like TCR9a is required to initiate tumor clearance. Subsequently, the other TCRs with lower affinities can engage the tumor and maintain tumor clearance, while not suffering activation induced cell death like the T cells bearing high affinity receptors. Namely, it may be necessary to select oligoclonal T cells with varying tumor affinities to balance the need for effective tumor killing and persistence in circulation. An alternative interpretation is that TCR9a was not necessary for clinical efficacy, and the therapeutic effect was due to TCRs with lower affinities, namely TCR10 and TCR9b&c. Indeed, it appears that intermediate affinity TCRs with low micromolar to high nanomolar affinities such as TCR10, 9b and 9c, are ideal for effective TCR therapy (68–70). Further experiments will need to investigate the validity of this model, specifically whether infusions of multiple TCRs with different antigen specificities, affinities and capacities for *in vivo* persistence are all necessary for effective adoptive T cell therapy. It is worth noting that the literature on high affinity TCRs is somewhat confused, in part due to the use of affinity by some investigators in place of avidity and a lack of consistency regarding what is meant by ‘high affinity’ (74). As an example, this 2012 study concluded that high affinity TCRs exhibited improved cytotoxicity, survival and reduced expression of inhibitory receptors (74). However, the affinity of the TCRs were not defined, and merely compared two TCRs with differing avidity measured by tetramer staining in a previous study (75). Both studies use affinity, when avidity is appropriate as direct affinity measurements were not made, and it is not clear where the affinities of the two TCRs lie on a scale from high micromolar to low nanomolar. For our part, we consider low affinity TCRs to have a K_D_ > 10 μM, intermediate, K_D_ = 100 nM – 10 μM, high, K_D_ = 1nM – 100 nM, and supraphysiological, K_D_ < 1nM.

In addition to antigenic affinity, the persistent expansion of TCR10 during adoptive transfer therapy highlights a potential need to include T cells with broader breadth in neoantigen recognition. In this case, TCR10 recognizes the KRAS-G12D 10mer peptide in a conformation not cross-reactive to the other three TCRs. It showed that the same oncogenic mutation may produce different conformational neoantigens that require non-cross-reactive T cell responses. Namely, the most potent T cell clones against one neoantigen may not be effective against other conformational variants of the same mutation-derived neoantigens and thus risking tumor escape. When taking both neoantigen variation and anti-tumor affinity into consideration, we propose a potentially more effective rational approach to screen TIL for adoptive T cell transfer therapy. Once the antigenic forms of a tumor antigen are defined, a combined structural and biochemical approach can be applied to select favorable therapeutic T cell clones with broad antigen affinities and specificities against variant neoantigens, both mutational and conformational.

## Conclusions

Previous studies classified neoantigens into at least two classes, those with mutations at anchor residues (group 2) and those with mutations in existing ‘self’ epitopes (group1) (32, 62–64). Recent structural studies have built on this work to provide unprecedented insight into how these structurally distinct classes of neoantigens are detected by T cells (15, 39–41). Recognition of neoantigens by T cells is an essential component of many successful immunotherapies (8). However, the biochemical and structural features of immunogenic neoantigens and effective therapeutic TCRs are still under investigation. While data are limited, there is evidence that TCRs specific for group 2 neoantigens can be of high affinity and this may explain in part why group 2 neoantigens are more immunogenic (62–64). The study of TCRs with demonstrated clinical efficacy revealed several traits associated with clinical success, such as a potential benefit of balancing between high affinity and high persistence. This multifaced requirement is best met with a rational approach in selecting TCRs with known biophysical characteristics for therapeutic use.

## Author Contributions

MS performed the experiments and wrote the manuscript. PS wrote the manuscript. All authors contributed to the article and approved the submitted version.

## Funding

The funding of this work is provided by the Division of Intramural Research, National Institute of Allergy and Infectious Diseases, National Institutes of Health.

## Author Disclaimer

The content of this publication does not necessarily reflect the views or policies of the Department of Health and Human Services, nor does mention of trade names, commercial products, or organizations imply endorsement by the U.S. Government.

## Conflict of Interest

The authors declare that the research was conducted in the absence of any commercial or financial relationships that could be construed as a potential conflict of interest.

## Publisher’s Note

All claims expressed in this article are solely those of the authors and do not necessarily represent those of their affiliated organizations, or those of the publisher, the editors and the reviewers. Any product that may be evaluated in this article, or claim that may be made by its manufacturer, is not guaranteed or endorsed by the publisher.
